# Supplementary material for: Effects of competition and ownership on the simultaneous relationship between bank risk and capital: Evidence from an emerging economy of Bangladesh
Source: PLoS One. 2024 Dec 6;19(12):e0311428. doi: 10.1371/journal.pone.0311428 (PMC11623798; doi:10.1371/journal.pone.0311428)
Supplement: S1 Appendix — (DOCX) [file pone.0311428.s001.docx]

**Appendix A:**

**Table A1. Granger Causality Test (GCT)**

| Null Hypothesis | Two lags | | Five lags | |
| --- | --- | --- | --- | --- |
|  | F-statistics | Probability | F-statistics | Probability |
| CAR does not Granger Cause NPL | 2.58857 | 0.0787* | 5.08512 | 0.0002*** |
| NPL does not Granger Cause CAR | 6.90451 | 0.0011*** | 2.91894 | 0.0139** |
| Note: The table displays the GCT. The asterisks represent different levels of significance, where *** indicates 1%, ** indicates 5%, and * denotes 10%. | | | | |

**Appendix B:**

**Table B1. Test for the cointegration (Capital Equation)**

| Particulars | Value | Decision |
| --- | --- | --- |
| **Westerlund test for Cointegration** | | |
| Variance Ratio | -2.8704*** | Ha: All the Panels are cointegrated  (Accepted) |
| Number of Panels | 44 |  |
| Avg Number of Periods | 11.227 |  |
| **Pedroni test for Cointegration** | | |
| Modified Phillips–Perron t | 6.2161*** | Ha: All the Panels are cointegrated  (Accepted) |
| Phillips–Perron t | -17.5102*** |  |
| Augmented Dickey–Fuller t | -15.3407*** |  |
| Number of Panels | 44 |  |
| Average Numbers of Periods | 10.227 |  |
| Augmented Lags | 1 |  |
| Lags: Newey–West | 2.00 |  |

Note: The asterisks represent different levels of significance, where *** indicates 1%, ** indicates 5%, and * denotes 10%.

**Table B2. Test for the cointegration (Risk Equation)**

| **Particulars** | **Value** | **Decision** |
| --- | --- | --- |
| **Westerlund test** | | |
| Variance Ratio | -2.4604*** | Ha: All the Panels are cointegrated  (Accepted) |
| Number of Panels | 44 |  |
| Avg Number of Periods | 11.227 |  |
| **Pedroni test** | | |
| Modified Phillips–Perron t | 7.3350*** | Ha: All the Panels are cointegrated  (Accepted) |
| Phillips–Perron t | -10.5537*** |  |
| Augmented Dickey–Fuller t | -13.5800 *** |  |
| Number of Panels | 44 |  |
| Average Numbers of Periods | 10.227 |  |
| Augmented Lags | 1 |  |
| Lags: Newey–West | 2.00 |  |

Note: The asterisks represent different levels of significance, where *** indicates 1%, ** indicates 5%, and * denotes 10%.

**Appendix C:**

**Table C1: Regressors Endogeneity Test**

| Dependent variable | Difference in J-stats | d.f. | Probability |
| --- | --- | --- | --- |
| Risk -NPLTL | 3.97495 | 1 | 0.0462 |
| Risk- with CAR^2^ | 7.852915 | 1 | 0.0051 |
| Capital -CAR | 93.82376 | 1 | 0.0000 |
| Capital with NPLTL^2^ | 119.3724 | 1 | 0.0000 |
| Note: Probability value signifies the endogeneity effect of endogenous variable over dependent variable. | | | |

| **Table C2: Breusch-Pagan test for heteroskedasticity** | | | | |
| --- | --- | --- | --- | --- |
|  | Dependent Variables | | | |
|  | Risk-NPLTL | Risk with CAR2 | Capital as CAR | Capital with NPLTL2 |
| Chi2 Value | 84.05 | 69.07 | 1320.14 | 1385.75 |
| Prob > chi2 | 0.000 | 0.000 | 0.000 | 0.000 |

Note: Significant probability value nullify the hypothesis “no heteroskedasticity”

| **Table C3: Breusch-Godfrey LM test for autocorrelation** | | | |
| --- | --- | --- | --- |
| Dependent variable | chi2 | df | Prob > chi2 |
| Risk-NPLTL | 234.613 | 1 | 0.000 |
| Risk with CAR2 | 256.538 | 1 | 0.000 |
| Capital- CAR | 27.917 | 1 | 0.000 |
| Capital with NPLTL2 | 29.686 | 1 | 0.000 |

Note: Significant probability value nullify the hypothesis “no serial correlation”

**Table C4: Hausman test for Fixed -Random effect.**

| Dependent variable |  | Prob > chi2 |
| --- | --- | --- |
| Risk-NPLTL |  | 0.000 |
| Risk with CAR2 |  | 0.000 |
| Capital- CAR |  | 0.000 |
| Capital with NPLTL2 |  | 0.000 |
| Note: Significant probability value refers the fixed effect. | | |
