# Supplementary material for: Effects of competition and ownership on the simultaneous relationship between bank risk and capital: Evidence from an emerging economy of Bangladesh
Source: PLoS One. 2024 Dec 6;19(12):e0311428. doi: 10.1371/journal.pone.0311428 (PMC11623798; doi:10.1371/journal.pone.0311428)
Supplement: Attachment — Submitted filename: Nitsuh Alemayehu reviewing paper.docx [file pone.0311428.s003.docx]

**Comments, Suggestions and Questions of the Article**

Thank you for inviting me on this interesting article as a peer reviewer for the publication. Here I have forwarded some recommendation, and question after a deep review of the article as follow:

| S/n | Topic/section | Comments, Suggestions and Questions |
| --- | --- | --- |
| 1. | Topic | The topic is Very interesting but it is better to state study area like  “Effects of competition and ownership on the simultaneous relationship between bank risk and capital: evidence from an emerging economy of Bangladesh”. |
| 2. | Abstract | 1. You did not mention the objective of the study, crucial background, So, clearly describe it. 2. You did not mention the research methodology that you used, so clearly state your research methodology that you used.   The reset section of the abstract is good. |
| 3. | Introduction | Your introduction sections are strong and you are clearly articulate the research problem and its relevance.  However, when you state your research gap you have stated only title gap and variable gap. So clearly mention the other relevant research gaps that you are interested to do this research topic (clear gap analysis and methods used by other studies) |
| 4. | Theoretical and Empirical literature review | 1. You are mentioning the empirical review of the previous researcher main finding and develop hypothesis, but you did not Clearly incorporate the variables that are used, the methodology they used in the empirical review section? 2. You have put dependent and independent variables under your variable description part, so it is better to put by using the conceptual framework to show its relationship. 3. You have described to use different theories under paragraph 5 of line 89 and 90 in your introduction part but, you are not discussing these theories in detail so, you should to discuss by relating to your research topic. 4. Your empirical literature review is not link with study area that is Bangladesh needed to be provided related literatures. |
| 5. | Methodology | 1. You did not mention the research design and sampling technique that you used. 2. You are not mentioning the model that you are used and the reason? 3. The rationale for the chosen time period 2010 to 2021 for data collection needs further explanation. Discuss how this period captures relevant economic cycles, regulatory changes, or other factors affecting computation and ownership of banks. |
| 6. | Empirical finding and Discussion | The discussion part is well discussed. You are discussed each independent variable relationship with the independent variable, so it is good. |
| 7. | Conclusions | Your conclusion is somewhat good but, you should briefly state your thoughts, your main points from your analysis and interpretation. |
| 8. | Recommendation | You only give recommendation for policy makers, you forget to mention direction for future researcher, banks and others. |
| ***Reference…****It is very good. I have not seen a problem it is well citied and you have use different references.* | | |
