# Supplementary material for: Effects of competition and ownership on the simultaneous relationship between bank risk and capital: Evidence from an emerging economy of Bangladesh
Source: PLoS One. 2024 Dec 6;19(12):e0311428. doi: 10.1371/journal.pone.0311428 (PMC11623798; doi:10.1371/journal.pone.0311428)
Supplement: Attachment — Submitted filename: Comments PONE-D-24-05802_reviewer.docx [file pone.0311428.s004.docx]

Reviewer 1 Comments:

The paper mentioned above is a good effort by the researcher(s); however, the following points need consideration.

1) The abstract is rather technical, please try to explain clearly. The findings in the abstract need to be written in a more informative way.
2) Motivation and contribution need to be strengthened. The contribution of this study needs more logical detail. The contribution of this paper is not well explained in the current version of the paper.
3) The authors should reorganize the structure of the introduction section to thoroughly express the aspects of this study including the background, current progress, motivation, the research question, the objective, the contribution, etc.
4) The introduction of the study is too short, even references are not enough to justify the topic. Please follow and cite some recent research papers such as;
   I. <https://doi.org/10.1016/j.jup.2024.101719>
   II. <https://doi.org/10.1007/s10614-022-10353-4>
   III. [https://doi.org/10.1016/j.egyr.2022. 11.054 2352-4847](https://doi.org/10.1016/j.egyr.2022.%2011.054%202352-4847)
   IV. <https://doi.org/10.1016/j.renene.2023.03.090>
   V. <https://doi.org/10.1007/s10668-023-03843-4>
5) The literature review is so lengthy unnecessary studies could be removed. Moreover, the manuscript could be substantially improved by relying and citing more on recent literature 2022-2023 such as the followings:
   i. <https://doi.org/10.1007/s10644-021-09321-z>
   ii. <https://doi.org/10.1016/j.esr.2022.1008>
   iii. <https://doi.org/10.1007/s10668-023-03413-8>
6) All equations need appropriate references in the text. Equation 2. is not appropriately referenced in the text.
7) Include the mean value of VIF in the last row in the Table. 3.
8) Divide your article into clearly defined and numbered sections. Subsections should be numbered 1.1 (then 1.1.1, 1.1.2, ...), 1.2, etc.
9) In the conclusion part, the authors should list all the limitations and discuss possible directions for further research.
10) Please check the manuscript again for errors.
