# Supplementary material for: Effects of competition and ownership on the simultaneous relationship between bank risk and capital: Evidence from an emerging economy of Bangladesh
Source: PLoS One. 2024 Dec 6;19(12):e0311428. doi: 10.1371/journal.pone.0311428 (PMC11623798; doi:10.1371/journal.pone.0311428)
Supplement: Attachment — Submitted filename: Review.docx [file pone.0311428.s005.docx]

Title: Effects of competition and ownership on the simultaneous relationship between bank risk and capital: evidence from an emerging economy

- The research results in the *Abstract* should present the specific relationship between bank capital and risk.
- Hypothesis 1 and 2 should clearly state the bidirectional relationship between bank risk and capital as considered in this article.
- The chapter on *Theoretical and Conceptual Background* can be appropriately simplified as part of the *Introduction* and *Literature review*.
- Date: The data should show how many state-owned, private, traditional, and Islamic banks each have as samples.
- The reasons for the inconsistency between the empirical results of this article and existing literature research should be explained, such as line 599.
- The regression results in each table should be reported as standard errors rather than P-values.
