# Supplementary material for: Effects of competition and ownership on the simultaneous relationship between bank risk and capital: Evidence from an emerging economy of Bangladesh
Source: PLoS One. 2024 Dec 6;19(12):e0311428. doi: 10.1371/journal.pone.0311428 (PMC11623798; doi:10.1371/journal.pone.0311428)
Supplement: Attachment — Submitted filename: Response to Reviewers.docx [file pone.0311428.s006.docx]

**Original Manuscript ID:** *PONE-D-24-05802*

**Original Article Title:** "*Effects of competition and ownership on the simultaneous relationship between bank risk and capital: evidence from an emerging economy*"

***General comments from the Honorable Editor***

Dear Dr. Chowdhury,

Thank you for submitting your manuscript to PLOS ONE. After careful consideration, we feel that it has merit but does not fully meet PLOS ONE’s publication criteria as it currently stands. Therefore, we invite you to submit a revised version of the manuscript that addresses the points raised during the review process.

***Response to Honorable Editor:***

Dear Sir, Thank you very much for allowing us to address your valuable comments and those of the honorable reviewers on our research paper. We have addressed the comments and attached the revised paper for your kind consideration.

Thank you very much for your attention and consideration.

Kind Regards,

The Authors

**Response to Editor**

General Comments:

Thank you for submitting your manuscript to PLOS ONE. After careful consideration, we feel that it has merit but does not fully meet PLOS ONE’s publication criteria as it currently stands. Therefore, we invite you to submit a revised version of the manuscript that addresses the points raised during the review process.

Authors' Response: *The authors thank the honorable reviewer for the positive and valuable comments. All the suggestions and comments are thoughtfully considered and addressed in the revision.*

| Journal Requirements | Authors’ response |
| --- | --- |
| Comment 1:  When submitting your revision, we need you to address these additional requirements.  Please ensure that your manuscript meets PLOS ONE's style requirements, including those for file naming. The PLOS ONE style templates can be found at  <https://journals.plos.org/plosone/s/file?id=wjVg/PLOSOne_formatting_sample_main_body.pdf> and  <https://journals.plos.org/plosone/s/file?id=ba62/PLOSOne_formatting_sample_title_authors_affiliations.pdf> | *Honorable Editor, thank you for your guidance. We have carefully reviewed and ensured that our manuscript adheres to PLOS ONE’s style requirements, including proper file naming and formatting as outlined in the provided templates.* |
| Comment 2: In the online submission form, you indicated that Data will be made available on request.  All PLOS journals now require all data underlying the findings described in their manuscript to be freely available to other researchers, either a. In a public repository, b. Within the manuscript itself, or c. Uploaded as supplementary information.  This policy applies to all data except where public deposition would breach compliance with the protocol approved by your research ethics board. If your data cannot be made publicly available for ethical or legal reasons (e.g., public availability would compromise patient privacy), please explain your reasons on resubmission and your exemption request will be escalated for approval. | *Dear Editor, thank you for the clarification regarding data availability. In compliance with PLOS ONE’s requirements, we have made all data available to other researcher.* |
| Comment 3: We note that you have referenced Berger, A.N., Ö. Öztekin, and R.A. Roman which has currently not yet been accepted for publication. Please remove this from your References and amend this to state in the body of your manuscript: (Berger, A.N., Ö. Öztekin, and R.A. Roman [Unpublished]) as detailed online in our guide for authors <http://journals.plos.org/plosone/s/submission-guidelines#loc-reference-style> | *Honorable Editor, thank you very much. We have updated the reference.* |
| Comment 4: f Please include captions for your Supporting Information files at the end of your manuscript, and update any in-text citations to match accordingly. Please see our Supporting Information guidelines for more information: <http://journals.plos.org/plosone/s/supporting-information>. | *Dear Editor, thank you for your guidance. We have included captions for all Supporting Information files at the end of the manuscript and updated the in-text citations to ensure they match accordingly. We have also reviewed the Supporting Information guidelines to ensure full compliance.* |

**Response to Reviewers:**

**Reviewer 1:**

**General Comments:**

Dear Editor,

From my point of view, it is a very interesting topic and simultaneously it seems that to the best of my knowledge is the first empirical study employs the two-step-system generalized method of moments to examine the effect of competition and ownership on the simultaneous association between risk and capital. The findings depict that competition and ownership significantly impact the relationship between risk and capital, which aligns with the moral hazard hypothesis, competition fragility hypothesis, and political view of state ownership.

The paper contains the following sections: Introduction, Literature Review, Data, Variables, and Methodology of the study, Empirical Research Framework, Empirical Findings and Discussions, and Conclusion. However, I find some recommendations.

**Authors' Response: *The authors thank the honorable reviewer for the positive and valuable comments. All the suggestions and comments are thoughtfully considered and addressed in the revision.***

| **Comments** | **Authors’ response** |
| --- | --- |
| Comment 1:  I suggest to the authors that the last section Conclusions and Policy Implications. | *Respected reviewer, thank you for your insightful observation. Following your observation, we renamed the conclusion “Conclusions and Policy Implications”.* |
| Comment 2: The abstract must contain the main purpose of the paper, the research method used in the research and the main contributions. | *Dear Reviewer, Thank you for your valuable feedback on our manuscript. We have carefully considered your comments and made the necessary revisions to the abstract to better align with your suggestions. Specifically, we have ensured that the abstract clearly outlines the paper's main purpose, the research methods employed, and the primary contributions of our study. Please see the abstract. These changes effectively address your concerns and enhance the clarity and comprehensiveness of our abstract. Thank you again for your insightful feedback, which has significantly improved the quality of our manuscript.* |
| Comment 3: It would be very useful to add the "Introduction" section and the purpose, objectives and hypothesis of the research. I consider that a weak point of the paper is that the authors did not show the novelty of the paper compared to other works. That is why, I consider that the introduction should specify the novelty of the paper compared to other papers published in this area. | *Dear revered reviewer, thank you very much. Following your comment, we add the purpose and objectives of the research in the introduction section. Please see, lines 78-80. We specify the hypotheses of the research in the literature review part. Moreover, we specify the novelty of the paper in the introduction part of the paper. Please see, lines 82-89.* |
| Comment 4: The research is well based on science and the results are in agreement with the theoretical part. From my point of view, the paper is original and the topic addressed brings added value to the specialized literature regarding financial markets. The paper is well written and easy to read. | *Dear valued reviewer, thank you very much for your insightful comments and positive feedback on our research paper. We sincerely appreciate your recognition of the alignment between our research findings and the theoretical underpinnings and your acknowledgment of the paper's originality and contribution to the specialized literature on financial markets. It is encouraging to hear that you found the paper well-written and easy to read, as we aimed to ensure clarity and accessibility for our readers. We are grateful for your thoughtful assessment.* |
| Comment 5: The research is well based on science and the results are in agreement with the theoretical part. The model applied to the analyzed data is correctly used in the analysis undertaken, it is a strength point of this paper. | *Thank you for your positive assessment of our research methodology and the alignment of our results with theoretical expectations. We are pleased to hear that you found the application of our analytical model appropriate and consider it a strength of the paper. Your feedback is valuable to us.* |
| Comment 6: The authors must specify the software used (STATA, Eviews, SPSS, etc.). Then the authors must do the Hausman test to detect the cross section with fixed or random effects. It must show the results of the Likelihood test, the heteroskedasticity test, and the endogeneity test. | *Dear reviewer, thank you for your nice observations and comments. Following your observation, we specify which software we used for data analysis in the study. Please see the Empirical research framework section (lines 416-417). We also show the heteroscedasticity test, and the endogeneity test. Please see Appendix C.* |
| Comment 7: The authors do not present the working hypotheses and do not use the standardized mathematical formulas for the econometric equations. | *Dear reviewer, thank you very much. As per your valuable suggestions, we have presented the working hypothesis (please see hypotheses 1 to 5) using the standardized mathematical formulas for the econometric equations Please see equations 2 to 11.* |
| Comment 8: It is very important for the authors to analyze the descriptive analysis (with Kurtosis test, Jarque Berra test and interpretation, Skewness and Kurtosis interpretation). In the same time the correlation analysis and the VIF test are very important in this research. | *Dear Reviewer, Thank you very much for your insightful suggestions. Following your comment, we analyze the descriptive statistics with skewness, kurtosis, and the Jarque Berra Test with interpretation. Please see Table 2 and Line numbers 470 – 474..* |
| Comment 9: Obviously, the application of a model must take into account the tests of heteroskedasticity, endogeneity etc. These aspects do not emerge from the method and results. In the same time, in the Robustness Checks section the authors have to apply an econometric method like regression or panel with fixed effect estimation or the random effect estimation (see for instance, Baltagi (2008), Hsiao (2014) and Andre B et al. (2015)). Besides, the corresponding tests to determine which is the best method of estimation is needed (see the Hausman test, the Breusch and Pagan (1980)´s Lagrange multiplier, the F test for fixed effects to test whether all unobservable individual effects are zero). | *We consider the multicollinearity tests (VIF) and white test for homoscedasticity for every model.*  *Our diagnosis results suggested fixed effects in the regression model (please see Table C4 in Appendix C). we opt for model regression with fixed effects in both the baseline model and robust check. We also run the heteroscedasticity test, endogeneity test, and the Breusch and Pagan Lagrange multiplier test (please see Appendix C).* |
| Comment 10: The authors talk about the relationship between these variables, however they do not support the empirical evidence providing panel cointegration tests that are crucial (see for instance Kao (1999) panel data cointegration test, the Pedroni (1999, 2004) panel data cointegration test or the Westerlund (2005) panel data cointegration test, among others. | *Dear Reviewer, Thank you very much. Following your valuable comments, we incorporated panel cointegration tests. Please see Lines 494 – 500, and also Tables B1 and B2 in Appendix B.* |
| Comment 11: The authors must show which are the instruments used by the 2SLS method. At the same time, the authors must explain when the null hypotheses of heteroskedasticity and endogeneity are accepted and what are the tests used in this regard. | *Dear Reviewer, following the suggestion we mentioned about the instrument variables in robust check. The diagnosis tests are also incorporated with the explanation of relevant hypotheses in Appendix C.* |
| Comment 12: I recommend the authors to refer to other recent works indexed in Web of Science, because only some cited works is not enough for a scientific paper. In my opinion, the authors must cite other papers regarding this subject and other subjects such as: tax compliance, banking, economic growth etc. That is why, I suggest that the authors cite papers published in Web of Science Journals, such as:  1. Batrancea, L., Rathnaswamy, M.K., & Batrancea, I. (2021). A Panel Data Analysis on Determinants of Economic Growth in Seven Non-BCBS Countries. Journal of the Knowledge Economy, 13, 1651–1665. 2. Batrancea, L., Rathnaswamy, M.M., Rus, M.S., & Tulai, H. (2022). Determinants of Economic Growth for the Last Half of Century: A Panel Data Analysis on 50 Countries. Journal of the Knowledge Economy. 3. Balcı, M.A., Batrancea, L.M., Akgüller, Ö., & Nichita, A. (2022). Coarse Graining on Financial Correlation Networks. Mathematics, 10(12), 2118. 4. Balcı, M.A., Batrancea, L.M., Akgüller, Ö., Gaban, L., Rus, M.-I., & Tulai, H. (2022). Fractality of Borsa Istanbul during the COVID-19 Pandemic. Mathematics, 10(14), 2503. 5. Batrancea, I., Batrancea, L., Rathnaswamy, M.M., Tulai, H., Fatacean, G., & Rus, M.-I. (2020). Greening the Financial System in USA, Canada and Brazil: A Panel Data Analysis. Mathematics, 8(12), 2217. 6. Batrancea, L. (2021). An Econometric Approach on Performance, Assets, and Liabilities in a Sample of Banks from Europe, Israel, United States of America, and Canada. Mathematics, 9(24), 3178. 7. Batrancea, L.M. Determinants of Economic Growth across the European Union: A Panel Data Analysis on Small and Medium Enterprises. Sustainability 2022, 14, 4797. <https://doi.org/10.3390/su14084797>. 8. Batrancea, L.M.; Balcı, M.A.; Chermezan, L.; Akgüller, Ö.; Masca, E.S.; Gaban, L. Sources of SMEs Financing and Their Impact on Economic Growth across the European Union: Insights from a Panel Data Study Spanning Sixteen Years. Sustainability 2022, 14, 15318. <https://doi.org/10.3390/su142215318> 9. Batrancea, L.M.; Balcı, M.A.; Akgüller, Ö.; Gaban, L. What Drives Economic Growth across European Countries? A Multimodal Approach. Mathematics 2022, 10, 3660. <https://doi.org/10.3390/math10193660>. 10. Batrancea, L.; Pop, M.C.; Rathnaswamy, M.M.; Batrancea, I.; Rus, M.-I. An Empirical Investigation on the Transition Process toward a Green Economy. Sustainability 2021, 13, 13151. <https://doi.org/10.3390/su132313151> 11. Batrancea, L., Batrancea, I., & Moscviciov, A. (2009). The Roots of the World Financial Crisis. Annals of the University of Oradea. Economic Sciences, 3, 57–62. 12. Moscviciov, A., Batrancea, I., Batrancea, M., Batrancea, L. (2010) Financial Ratio Analysis Used In The It Enterprises, Annals of Faculty of Economics, 1(2), 600-603. In conclusion, the article should be improve. It should also be enhanced with a review of the literature adequate to the subject and a broader interpretation and commentary of the research results. | *Dear Reviewer, thank you for your thoughtful feedback and for providing a list of recent publications from Web of Science journals. We appreciate your suggestion to broaden the scope of our article by citing additional relevant works. While we acknowledge the importance of referencing recent research in the field, we have carefully considered the suggested articles in relation to the focus and scope of our study. While some of the suggested papers are indeed valuable contributions to the literature, we have chosen to prioritize citations that directly align with the specific objectives and context of our research.*  *We have endeavored to ensure that our literature review encompasses a thorough examination of pertinent literature, including recent publications that substantiate and enrich our study. The selected citations are integral to supporting our arguments and contributing to the scholarly discourse. We sincerely appreciate your insights and suggestions, which have helped strengthen the quality and accuracy of our paper.* |

**Reviewer 2:**

**General Comments:**

Overall, the paper is an interesting read and has considerable scope. By considering the feedback provided and using the recommended references, the authors can enhance the study's impact and attract more interest. In short, I see this paper has potential to be considered for publication after incorporating the following suggestions.

**Authors' Response: *The authors thank the honorable reviewer for the positive and valuable comments. All the suggestions and comments are thoughtfully considered and addressed in the revision.***

| **Comments** | **Authors’ response** |
| --- | --- |
| Comment 1: Main Concern  I suggest the author add separate paragraphs of your study contribution citing papers to support their claim. I suggest that the contribution should be rewritten. In the current versions, there are no strong contributions. You should add here some details on the contribution of this paper as compared to previous literature and how it is different. In study contribution author(s) should cite past papers to support their claim. Example: <https://doi.org/10.1016/j.ribaf.2024.102305> This article contributes to the related literature in the following ways. First, according to the best of our knowledge, this study provides new cross-country evidence by exploring the non-linear patterns of ESG performance on sustainable growth rate of listed firms in the United States and China. This study is different from previous studies, such as (Chai et al., 2023, Fuente et al., 2022, Teng et al., 2021) adds a novel perspective to the existing body of research. Second, we analyze the non-linear effect of individual components on environmental, social, and governance performance and their distinct impact on sustainable growth. Third, we utilize the widely recognized measures of firms' sustainable growth rate models namely Robert Higgins widely employed in the domains of strategic management and corporate planning. Methodologically, we apply High Dimension Fixed Effects, feasible generalized least square, and dynamic panel two-stage generalized method of moments models to analyze the data. In addition, heterogeneous subgroups, and alternative model SG were developed by Van Horn. Furthermore, the exploration of the threshold effect of overall ESG performance, coupled with the analysis of individual environmental, social, and governance performance, introduces a fresh dimension to academic discussions. | *Honorable Reviewer, thank you for your guidance on how to improve the contribution section of our paper. We have carefully revised this section to address your suggestions and provide a clearer outline of our study's unique contributions. In the updated contribution section, located at Lines 84 -100 in the introduction part of the paper, we have incorporated separate paragraphs to highlight specific aspects of our research contribution, supported by citations to relevant past literature. These revisions emphasize the distinctiveness of our study compared to previous research in the field, offering a more comprehensive understanding of our contributions.* |
| Comment 2: Literature Review:  The following papers will help you in building theoretical farmwork, motivation, and in gap justification. Authors must improve the quality of references and add recent articles, such as.  <https://doi.org/10.1016/j.ribaf.2024.102305> <https://doi.org/10.1016/j.heliyon.2024.e26757> <https://doi.org/10.1016/j.bir.2024.01.002> <https://doi.org/10.24136/oc.2023.036> • This paper lacks a good knowledge of the key theories. Also, it is important to show the implications of these theories for your study. • I suggest authors to conclude literature section follow Example:  “Drawing from the study literature analysis, we believe that the interplay between ESG performance, traditional firm characteristics, industry dynamics, macroeconomic factors, and financial determinants in shaping corporate capital structure finds theoretical support from various established theories in corporate finance. In analyzing corporate capital structure in BRICS economies, several theories provide theoretical underpinnings and support. The trade-off theory suggests that firms balance the benefits of debt financing, such as tax shields, against the costs, such as bankruptcy risk, to determine an optimal capital structure (Miller & Modigliani, 1961). Agency theory highlights the conflicts of interest between stakeholders and managers, leading to the monitoring and disciplining role of debt in aligning interests (Meckling & Jensen, 1976). The pecking order theory posits that firms prefer internal financing over external debt due to information asymmetry, followed by debt over equity issuance(Myers, 1984a). Additionally, stakeholder theory emphasizes the importance of considering the interests of all stakeholders, including environmental and social concerns, in financial decision-making (Freeman, 1984). These theories provide a framework for understanding how ESG factors, firm characteristics, industry dynamics, macroeconomic conditions, and financial factors collectively influence corporate capital structure in BRICS economies. Drawing on well-established theories in corporate finance, such as stakeholder theory, TOT and POT , the study conceptual framework has been visualized in Figure 1” | *Dear esteemed revievewer, thank your for the nice comments. Following your comments we include the theoretical and conceptual background of the study, please see section 2.1.*  *We also conclude the literature review section by following your insightful observations. Moreover, we also develop the the conceptual frameworks, please see figure number 1. Furhtermore, we also improve the quality of the references and add recent articles by following your insightful observations.* |
| Comment 3: Data and Methodology  • In the current section data snad Sample constraction detial is complely missing ? I suggest authors to add one full set infirmation about data sourcses and period of smaple data and sample criteria. I am suggesting following papers to follow how to write this section <https://doi.org/10.1016/j.ribaf.2024.102305> <https://doi.org/10.1016/j.heliyon.2024.e26757> <https://doi.org/10.1016/j.bir.2024.01.002> <https://doi.org/10.24136/oc.2023.036> | *Honorable Reviewer, thank you for your valuable feedback on our manuscript. We have carefully considered your comments regarding the Data and Methodology. Specifically, we have addressed the concern about the lack of detailed information on data sources, the period of sample data, and the sample criteria. In response, we have revised the section to include comprehensive details about the data collection process, sources, and the criteria used for sample selection. We ensured the methodology aligned with the standards the referenced papers suggested. Thank you once again for your insightful feedback, which has significantly improved the quality of our manuscript.* |
| Comment 4: I have a concern regarding the explanation of the concept of "Inflection Point" as mentioned in line 403. While the formula provided sheds some light on the concept, I believe that a more thorough explanation is necessary to ensure clarity for the readers. Simply presenting the formula may not fully serve the purpose of elucidating the concept. I would suggest expanding upon the explanation of the "Inflection Point," providing more context and possibly offering examples to illustrate its significance within the context of your study. | *Dear Reviewer, thank you for your insightful feedback on explaining the "Inflection Point" concept. We have expanded the explanation in our manuscript to provide a more thorough understanding. We included a detailed definition, mathematical context, and practical significance of the inflection point within our study. Thank you again for your valuable suggestions.* |
| Comment 5: Empirical Results  • In my opinion, the authors should provide a more detailed discussion and provide economic justification /connectivity of findings. Drawing from past empirical studies and theoretical frameworks would not only enrich their analysis but also lend credibility to their arguments. | *Dear Reviewer, Thank you for this valuable suggestion. We have expanded the discussion section to provide a more detailed economic justification of our findings. We have also connected our results to relevant empirical studies, enriching the analysis and strengthening the credibility of our arguments.* |
| Comment 6: Conclusion  • Policy Implication should be improved. The authors should add more theoretical contributions and implications for managerial practice. These discussions could be more specific and thoughtful (take some practical examples).  • Add study limitations based on your study and at least one open research question for future researchers. | *Honorable Reviewer, thank you very much for your insightful suggestions. In response, we have significantly enhanced the policy implications section by providing more detailed and specific theoretical contributions and practical implications for managerial practice. Additionally, we also discuss the constraints of our research. We have also proposed an open research question that could guide future studies, helping to advance the field further.* |
| Comment 7: On a final note, I would recommend language editing. The references should be presented according to journal guidelines. In most of the reference volumes, the page number and issue are missing. | *Honorable Reviewer, thank you for your careful review. We have thoroughly revised the manuscript to improve the language and clarity, including a professional language edit. Additionally, we have carefully reviewed and updated the references to ensure they adhere to the journal's guidelines.* |
| Comment 8: Ensure that each table has explanatory notes to make it self-explanatory. This will improve the clarity and accessibility of the presented data for readers. | *Dear Reviewer, thank you for the valuable suggestion. We have added explanatory notes to each table in the manuscript to ensure they are self-explanatory. These notes clarify the data presented, making the tables more accessible and enhancing the overall clarity for readers.* |

**Reviewer 3:**

**General Comments:**

This paper addresses the important topic of the effects of competition and ownership on the relation between bank risk and capital in Bangladesh and has the merit of addressing and emerging country often neglected in literature, therefore filling an important research gap. However, some improvements might be needed to meet the journal standards.

**Authors' Response: *The authors thank the honorable reviewer for the positive and valuable comments. All the suggestions and comments are thoughtfully considered and addressed in the revision.***

| **Comments** | **Authors’ response** |
| --- | --- |
| Comment 1: To provide a comprehensive description of the data the skewness, kurtosis and Jarque-Bera test should also be addressed. | *Dear Reviewer, thank you very much for your valuable feedback regarding the need to comprehensively describe the data by including skewness, kurtosis, and the Jarque-Bera test. We have now included a detailed data distribution analysis addressing these issues. Thank you once again for your insightful feedback. We believe these additions have significantly strengthened our manuscript. Please see Table 2.* |
| Comment 2: Authors should perform autocorrelation and heteroskedasticity tests, which are missing in the paper and are important to examine the adequacy of the model estimates. | *Dear Reviewer, thank you very much for your valuable feedback. Considering your valuable comments, we showed the autocorrelation and heteroscedasticity tests. Please see Appendix C.* |
| Comment 3: As authors provide the estimates of ten models it would be interesting to understand how to discriminate between them. What is the best model to describe the data. | *Our equation 2 describes the baseline equation that represents the data. Other equations are extensions of equation 2. However, a detailed estimation of all equations is explained on page 11 (lines 411 to 416)* |
| Comment 4: finally, the residual analysis is not provided. Therefore, I suggest that it should be included in the paper. | *Respected reviewer, thank you very much for your valuable feedback. Considering your valuable feedback, we included the residual analysis in the paper. Please check tables 5 to 16; and Appendix A, B, and C.* |

**Reviewer 4:**

**General Comments:**

**Authors' Response: *The authors thank the honorable reviewer for the positive and valuable comments. All the suggestions and comments are thoughtfully considered and addressed in the revision.***

| **Comments** | **Authors’ response** |
| --- | --- |
| Comment 1: Topic  The topic is Very interesting but it is better to state study area like “Effects of competition and ownership on the simultaneous relationship between bank risk and capital: evidence from an emerging economy of Bangladesh”. | *Honorable reviewer, thank you for your insightful feedback. Following your comments, we renamed the study “Effects of competition and ownership on the simultaneous relationship between bank risk and capital: evidence from an emerging economy of Bangladesh.”* |
| Comment 2: Abstract  1.You did not mention the objective of the study, crucial background, So, clearly describe it.  2.You did not mention the research methodology that you used, so clearly state your research methodology that you used. The reset section of the abstract is good. | *Honorable Reviewer, Thank you very much for your insightful feedback. We have revised the abstract to clearly describe the study's objective and provide crucial background information. Additionally, we have explicitly mentioned the research methodology used in our study. The revised abstract now clearly states our aim to investigate the simultaneous relationship between bank risk and capital, and it includes background on the joint influence of competition and ownership on this relationship. It also specifies that we employed the two-step system generalized method of moments. These revisions aim to clarify the study's purpose, context, and methodology, addressing your comments effectively.* |
| Comment 3: Introduction  Your introduction sections are strong and you are clearly articulate the research problem and its relevance. However, when you state your research gap you have stated only title gap and variable gap. So clearly mention the other relevant research gaps that you are interested to do this research topic (clear gap analysis and methods used by other studies) | *Honorable Reviewer, Thank you for your insightful feedback regarding identifying and articulating research gaps in our manuscript. Based on your suggestion, we have revised the Introduction section to include a more comprehensive and detailed gap analysis (Please see lines 65-80). Thank you again for your valuable comments and suggestions.* |
| Comment 4: Theoretical and Empirical Literature Review  1.You are mentioning the empirical review of the previous researcher main finding and develop hypothesis, but you did not Clearly incorporate the variables that are used, the methodology they used in the empirical review section?  2.You have put dependent and independent variables under your variable description part, so it is better to put by using the conceptual framework to show its relationship. 3.You have described to use different theories under paragraph 5 of line 89 and 90 in your introduction part but, you are not discussing these theories in detail so, you should to discuss by relating to your research topic.  4.Your empirical literature review is not link with study area that is Bangladesh needed to be provided related literatures. | *Respected reviewer, thank you very much for your valuable observations. We followed your comments and address the comments:*  *(1)* *Thank you for your valuable feedback regarding the empirical review section. Based on your suggestion, we have revised this section to incorporate the variables and methodologies used in the previous studies we reviewed. Thank you again for your insightful comments. Please see sections 2.2.1, 2.2.2, and 2.2.3.*  *(2) We show the relationship between the variables in the conceptual framework. Please see Figure number 1.*  *(3) Following your comments we detailed the theories as the “theoretical and conceptual background. Please see section 2.1.*  *(4)* *Thank you for highlighting the need to contextualize the empirical literature review with studies related to Bangladesh. we have revised the literature review section to incorporate relevant studies that specifically address the banking sector, capital regulations, and risk-taking behaviors within the Bangladeshi context. Please see sections 2.2.1, 2.2.2, and 2.2.3..* |
| Comment 5: Methodology  1.You did not mention the research design and sampling technique that you used.  2. You are not mentioning the model that you are used and the reason?  3.The rationale for the chosen time period 2010 to 2021 for data collection needs further explanation. Discuss how this period captures relevant economic cycles, regulatory changes, or other factors affecting computation and ownership of banks. | *(1) we opt for the quantitative research design mentioned on page 7 (lines 289 and 290). We incorporate all available data. Only nonavailable data and data with consecutive missing figures for more than five years are excluded from the data set.*  *(2) The model used in the study with the necessary support of using the model is given in section 3.3, page 9 (lines 328 to 340)*  *(3) Honorable reviewer, following your comments, we incorporate the rationale for the chosen time period in the Data section. Please see lines 282-288 (section 3.1.* |
| Comment 6: Empirical Findings and Discussion  The discussion part is well discussed. You are discussed each independent variable relationship with the independent variable, so it is good. | *Dear Reviewer, Thank you for your positive feedback regarding the discussion section of our manuscript. We are pleased to hear that our detailed examination of each independent variable's relationship with the dependent variable was well-received. Your encouraging words motivate us to strive for clarity and thoroughness in our research.* |
| Comment 7: Conclusion  Your conclusion is somewhat good but, you should briefly state your thoughts, your main points from your analysis and interpretation. | *Dear Reviewer, Thank you for this valuable suggestion. We have revised the conclusion to succinctly summarize our key findings and main points from the analysis and interpretation. This revision aims to provide a clearer and more focused summary of our study’s contributions.* |
| Comment 8: Recommendation  You only give recommendation for policy makers, you forget to mention direction for future researcher, banks and others. | *Respected Reviewer, thank you for pointing out the need to provide recommendations for policymakers, future researchers, banks, and other stakeholders. Based on your suggestions, we have revised the conclusion and recommendations section of the manuscript to address this issue comprehensively. Please see lines 813-837.* |
| Comment 9: Reference  It is very good. I have not seen a problem it is well citied and you have use different references. | *Dear Reviewer, Thank you for your positive feedback regarding our referencing. We are pleased to hear that you found our citations comprehensive and well-sourced. Your encouraging words motivate us to maintain high standards in our scholarly work.* |

**Reviewer 5:**

**General Comments:**

The paper mentioned above is a good effort by the researcher(s); however, the following points need consideration.

**Authors' Response: *The authors thank the honorable reviewer for the positive and valuable comments. All the suggestions and comments are thoughtfully considered and addressed in the revision.***

| **Comments** | **Authors’ response** |
| --- | --- |
| Comment 1: The abstract is rather technical, please try to explain clearly. The findings in the abstract need to be written in a more informative way. | *Respected Reviewer, thank you for your constructive feedback. We have revised the abstract to make it less technical and to present the findings in a more informative way. The revised abstract uses clearer language to explain our study's purpose, methods, and key findings. We have ensured that the presentation of results is more accessible and informative, highlighting our findings' significant relationships and implications. We believe these changes improve the readability and informativeness of the abstract, addressing your concerns.* |
| Comment 2: Motivation and contribution need to be strengthened. The contribution of this study needs more logical detail. The contribution of this paper is not well explained in the current version of the paper. | *Respected Reviewer, thank you very much for your valuable feedback. We have made significant efforts to strengthen the motivation and clarify the contributions of our study in the revised manuscript.* |
| Comment 3: The authors should reorganize the structure of the introduction section to thoroughly express the aspects of this study including the background, current progress, motivation, the research question, the objective, the contribution, etc. | *Dear Reviewer, Thank you for your insightful feedback on the introduction section of our manuscript. Based on your suggestions, we have reorganized the introduction to thoroughly express the aspects of our study, including the background, motivation, research questions, objectives, and contributions. We believe these changes enhance the clarity and comprehensiveness of our manuscript.* |
| Comment 4: The introduction of the study is too short, even references are not enough to justify the topic. Please follow and cite some recent research papers such as;  I. <https://doi.org/10.1016/j.jup.2024.101719> II. <https://doi.org/10.1007/s10614-022-10353-4> III. <https://doi.org/10.1016/j.egyr.2022>. 11.054 2352-4847 IV. <https://doi.org/10.1016/j.renene.2023.03.090> V. <https://doi.org/10.1007/s10668-023-03843-4> | *Respected Reviewer, Thank you for your constructive feedback regarding the introduction of our study. Based on your suggestion, we have expanded the Introduction section to provide a more comprehensive background and justification for our research. Thank you again for your valuable comments and suggestions.* |
| Comment 5: The literature review is so lengthy unnecessary studies could be removed. Moreover, the manuscript could be substantially improved by relying and citing more on recent literature 2022-2023 such as the followings:  i. <https://doi.org/10.1007/s10644-021-09321-z> ii. <https://doi.org/10.1016/j.esr.2022.1008> iii. <https://doi.org/10.1007/s10668-023-03413-8> | *Honorable Reviewer, Thank you for your valuable feedback on the literature review section of our manuscript. Based on your suggestions, we have streamlined the literature review by removing unnecessary studies, where necessary, to enhance clarity and focus. Additionally, we have incorporated recent literature, as you suggested. Thank you again for your insightful comments and suggestions.* |
| Comment 6: All equations need appropriate references in the text. Equation 2. is not appropriately referenced in the text. | *Honorable Reviewer, thank you for pointing this out. We have carefully reviewed the manuscript and ensured that all equations, including Equation 2, are now appropriately referenced in the text. We have added clear references and explanations where necessary to improve the flow and coherence of the mathematical content.* |
| Comment 7: Include the mean value of VIF in the last row in the Table. 3. | *Dear esteemed reviewer, thank you very much for your insightful observations. We include the mean value of VIF in the last row in Table 3. Please see the Table 3.* |
| Comment 8: Divide your article into clearly defined and numbered sections. Subsections should be numbered 1.1 (then 1.1.1, 1.1.2, ...), 1.2, etc. | *Dear Reviewer, thank you very much for your valuable suggestion regarding the article's structure. We have revised the manuscript to include clearly defined and numbered sections and subsections, following the recommended format (e.g., 1.1, 1.1.1, 1.2, etc.). This restructuring enhances the clarity and organization of the paper, making it easier for readers to follow the content.* |
| Comment 9: In the conclusion part, the authors should list all the limitations and discuss possible directions for further research. | *Dear Reviewer, thank you. Considering your valuable comments, we discuss the limitations of our research. We have also proposed an open research question that could guide future studies, helping to advance the field further.* |
| Comment 10: Please check the manuscript again for errors. | *Honorable Reviewer, Thank you for your feedback. We have thoroughly reviewed the manuscript and addressed any errors to ensure clarity and accuracy. We appreciate your attention to detail and believe the revised version reflects these improvements.* |

**Reviewer 6:**

**General Comments:**

**Authors' Response: *The authors thank the honorable reviewer for the positive and valuable comments. All the suggestions and comments are thoughtfully considered and addressed in the revision.***

| **Comments** | **Authors’ response** |
| --- | --- |
| Comment 1: The research results in the Abstract should present the specific relationship between bank capital and risk. | *Dear esteemed reviewer, thank you very much for your detailed feedback. We have revised the abstract to present the specific relationship between bank capital and risk more clearly. The revised abstract now explicitly details the significant non-linear bidirectional relationship between bank risk and capital, including how capital exhibits a U-shaped effect on risk and how risk has a non-linear negative impact on capital.* |
| Comment 2: Hypothesis 1 and 2 should clearly state the bidirectional relationship between bank risk and capital as considered in this article. | *Dear Reviewer, Thank you for your valuable feedback regarding the clarity of our hypotheses. Based on your suggestion, we have revised Hypotheses 1 and 2, combining them into a single hypothesis to clearly state the bidirectional relationship between bank risk and capital, as considered in our study (Please see hypothesis 1). The revised hypothesis now explicitly reflects both directions of this relationship, ensuring a more precise alignment with the focus of our research. Thank you again for your insightful comments.* |
| Comment 3: The chapter on Theoretical and Conceptual Background can be appropriately simplified as part of the Introduction and Literature review. | *Dear Reviewer, Thank you for your insightful feedback regarding the structure of our manuscript. Based on your suggestion, we have integrated the Theoretical and Conceptual Background chapter into the Literature Review parts. This consolidation aims to streamline the flow of the manuscript and provide a more cohesive narrative. The theoretical and conceptual background is now included before the empirical review and hypotheses development, ensuring a logical progression of ideas. Thank you again for your valuable input.* |
| Comment 4: Date: The data should show how many state-owned, private, traditional, and Islamic banks each have as samples. | *Dear Reviewer, thank you for your insightful observations. We have taken your feedback into consideration and have included the details regarding the number of state-owned, private, conventional, and Islamic banks included in the sample. Please refer to section 3.1 in the data section for this information.* |
| Comment 5: The reasons for the inconsistency between the empirical results of this article and existing literature research should be explained, such as line 599. | *Honorable Reviewer, thank you for your insightful comment. In response, we have added a detailed explanation in the relevant section to clarify the reasons for the inconsistency between our empirical results and those reported in the existing literature. We have considered factors such as differences in the study period, economic conditions, sample selection, and methodology, which may contribute to the observed discrepancies. This additional discussion aims to understand better the context in which our findings were obtained.* |
| Comment 6: The regression results in each table should be reported as standard errors rather than P-values. | *Following the reviewer's comments, we report the standard errors in respective tables. Please see Tables 5 -16.* |

**Reviewer 7:**

**General Comments:**

**Authors' Response: *The authors thank the honorable reviewer for the positive and valuable comments. All the suggestions and comments are thoughtfully considered and addressed in the revision.***

| **Comments** | **Authors’ response** |
| --- | --- |
| Comment 1: The names of the variables (i.e., “endo”) in some equations seem irrelevant and difficult to follow. The authors should change the notation of the variables. | *Honorable Reviewer, Thank you for your valuable feedback regarding the notation of the variables in our equations. We have revised the notation to enhance clarity and readability. Please refer to equations (2) to (9) for the updated notation in the revised manuscript. We believe this revised notation, along with the comprehensive explanations provided, will address your concerns and improve the overall clarity of our manuscript. Thank you again for your insightful comments.* |
| Comment 2: Equation 11 (inflection point) is not necessary. | *Respected Reviewer, Thank you for your valuable feedback. We deeply appreciate the time and effort you've dedicated to providing valuable insights to improve our work. However, one of the honorable reviewers suggested that we provide a more thorough explanation of the concept of the "inflection point" to ensure clarity for readers. Considering this recommendation, we have expanded on the concept of the inflection point, including additional context and practical significance within our study. We acknowledge that the other honorable reviewer expressed concerns regarding the necessity and suitability of including inflection points in the estimation results. Given the importance of addressing both perspectives, we have maintained our expanded explanation in the revised manuscript to enhance understanding and provide comprehensive insights.* |
| Comment 3: The description of variables in Table 1 should be described by words rather than by formula. | *Dear Reviewer, Thank you. Following your comment, we describe the formula in words rather than formula form. Please see Table 1.* |
| Comment 4: The inverse of b is 1/b, not -b. The author should correct the use of word. | *Dear Reviewer, Thank you for your insightful comment. Following your suggestion, we have revised the terminology used in the manuscript. Rather than referring to the "inverse" of BI, we now employ the term "opposite" to indicate the negation precisely. We are grateful for your constructive input, which has enhanced the clarity and precision of our presentation.* |
| Comment 5: The authors should check for typos and errors such as “by utilizing different specifications of the dependent and endogenous independent variables and the dependent and endogenous independent variables. | *Dear reviewer, Thank you so much for your keen observations. We have corrected these typo errors based on your observations.* |
| Comment 6: The authors should describe the instrumental variables in the robustness check in more details. | *Dear Reviewer, thank you very much. Following the suggestion we mentioned about the instrument variables in robust check (please see section 4.3).* |
| Comment 7: The research hypotheses should be better developed and revised. | *Dear valued reviewer, Thank you for your insightful feedback on the development of our research hypotheses. We have revisited and revised the hypotheses to ensure they are more clearly articulated and robustly developed. This enhancement aims to better align the hypotheses with our research objectives and provide a more explicit framework for our study. Thank you again for your valuable input.* |
| Comment 8: The inclusion of inflection points in the estimation results is not necessary and unsuitable. | *Respected Reviewer, Thank you for your valuable feedback. We deeply appreciate the time and effort you've dedicated to providing valuable insights to improve our work. However, one of the honorable reviewers suggested that we provide a more thorough explanation of the concept of the "inflection point" to ensure clarity for readers. Considering this recommendation, we have expanded on the concept of the inflection point, including additional context and practical significance within our study. We acknowledge that the other honorable reviewer expressed concerns regarding the necessity and suitability of including inflection points in the estimation results. Given the importance of addressing both perspectives, we have maintained our expanded explanation in the revised manuscript to enhance understanding and provide comprehensive insights.* |
| Comment 9: The references are not sorted and have considerable errors. | *Dear Respected Reviewer, thank you very much for your valuable observations regarding the references. We have carefully reviewed the referencing style and ensured it adheres to the guidelines suggested by the journal. We utilized the prescribed style from this link* [*https://endnote.com/downloads/styles/plos-public-library-of-science-all-journals/*](https://endnote.com/downloads/styles/plos-public-library-of-science-all-journals/)*. Furthermore, we have thoroughly checked for any errors and corrected them accordingly. We appreciate your feedback, which has helped us improve the quality of our manuscript. Thank you again for your insightful comments.* |
